# Identification and Characterization of the Transcriptional Regulator ChrB in the Chromate Resistance Determinant of *Ochrobactrum tritici* 5bvl1

**DOI:** 10.1371/journal.pone.0077987

**Published:** 2013-11-04

**Authors:** Rita Branco, Paula V. Morais

**Affiliations:** 1 IMAR-CMA-Marine and Environmental Research Centre, Coimbra, Portugal; 2 Interdisciplinary Research Institute, University of Coimbra, Coimbra, Portugal; 3 Department of Life Sciences, FCTUC, University of Coimbra, Coimbra, Portugal; Centre National de la Recherche Scientifique, Aix-Marseille Université, France

## Abstract

*Ochrobactrum tritici* 5bvl1 is able to resist to high concentrations of chromate through the expression of an inducible chromate-resistant determinant, found in a mobile element (Tn*OtChr*), which carries the genes, *chrB*, *chrA*, *chrC* and *chrF*. The regulation of *chr* operon present in Tn*OtChr*, which is controlled by a transcriptional regulator, ChrB, was characterized in the current work. Fusions of *chr* promoter, or *chr* promoter and *chrB* gene, upstream of a *gfp* reporter gene, identified the most probable promoter sequence within the *tnpR-chrB* intergenic region. This region contains an AT-rich imperfect inverted repeat sequence, which overlaps a part of the −10 sequence. The results of the *in vitro* DNA-binding assays with purified ChrB (His- or no-tagged) showed that the protein binds directly to the *chr* promoter region. In order to identify the ChrB functional domain for sensing chromate stress and for DNA-binding, site-directed mutagenesis of ChrB was performed. Among several single amino acid mutants, three mutants (R180; R187 and H229) prevented chromate induction without any modification to the protein’s stability. Interestingly, two ChrB mutants (R18 and R23) were constitutively active, regardless of chromate stress conditions, indicating that the residues most probably belong to the protein-DNA binding site. As such, the ChrB was classified as a transcriptional regulator that recognizes a specific DNA sequence, regulating the expression of a chromate resistance determinant.

## Introduction

Human activities have resulted in the release and introduction into the environment of different chemicals including heavy metals. In general, most metals are essential for microbial cells, as co-factors for different enzymes or structural components of proteins [1]. Nevertheless, many essential metals become toxic at high ion concentrations, while some metal ions are toxic to bacterial cells at any concentration. Therefore, the interest in discovering how bacteria are dealing with hazardous environmental pollutants resulted in numerous and important genetic, biochemical and physiological data, which allowed a deeper understanding of the adaptation capacities of microorganisms. Many bacteria contain genes that encode specific products conferring resistance to heavy metal ions. Some of the proteins that have been characterized are membrane-bound transporters that pump toxic ions out of the cells, cytoplasmic or periplasmic metal transport proteins, metal reductases, metallothioneins and metal-sequestrating proteins [2], [3]. In most microorganisms, the expression of such resistance systems is controlled at transcription level by metal sensor proteins, which are known to bind to the promoter regions and are responsible for regulation of metal responsiveness.

The biodegradation capabilities of microorganisms, with the purpose of applying these directly in bioremediation processes have been looked at with interest [4]. In the past, less attention was endorsed to studies of the regulatory mechanisms, which manage the expression of specific pathways. Nowadays, the particular regulation features of several kinds of expression pathways have attracted interest of numerous researchers [5]. Different groups, that studied microbial resistance mechanisms, also tried to discover which regulatory process is behind it and/or the exact mechanisms of activation and repression of the systems. Moreover, information about the regulation of systems has acquired a special importance, since these regulatory systems have the potential for being used as sensory mechanisms in the construction of bioreporters. These tools have been recognized as useful and very promising instruments in monitoring the quality of many environments, such as water, soil, and air [6]–[8].

Chromium ion is considered to be an important element on its reduced form [Cr (III)] but becomes toxic on the oxidised state of chromate or dichromate [9]. Therefore, the intracellular amount of chromium ions must be tightly regulated to prevent high chromate concentrations. Several chromate resistance determinants were identified in prokaryotes and among the reported bacterial systems are those of: *Cupriavidus metallidurans* CH34 [10], *Pseudomonas aeruginosa* PAO1 [11], *Shewanella* sp. strain ANA-2 [12], *Synechococcus elongatus* PCC 7942 [13], *Lysinibacillus fusiformis* ZC1 [14], *Bacillus cereus* SJ1 [15], *Arthrobacter* sp. strain FB24 [16] and *Ochrobactrum tritici* 5bvl1 [17]. Chromate resistance in bacteria is primarily accomplished by a specific efflux system that pumps chromate out of the cell, thereby lowering the intracellular concentration [17]–[19]. This function is performed by ChrA transporter, which is a *chr* operon encoded protein. Besides *chrA*, other genes have also been identified in bacterial *chr* determinants, such as *chrB*, *chrC* and other less-studied genes (*chrE* or *chrF*). In a previous study, we identified a Tn*OtChr* element of chromate resistant strain *O. tritici* 5bvl1, carrying the *chr* operon that comprises *chrB*, *chrA*, *chrC* and *chrF* genes [20]. The *chrB* is proposed to play a regulatory role for expression of the ChrA transporter, *chrC* encodes a putative superoxide dismutase and *chrF* encodes a protein with uncertain function [21].

In the best of our knowledge no chromate responsive regulators have been well characterized or really studied. In this study, we aimed to identify the *chr* promoter region and clarify the role of ChrB. Several constructions performed by fusion of putative *chr* promoter fragments with the reporter gene *gfp* allowed to limit the most predictable *chr* promoter sequence. Several approaches, such as transcriptional fusion of *chrB* with *gfp*, electrophoretic mobility shift assay (EMSA) and site directed mutagenesis were also performed to characterize the ChrB protein, its binding to the promoter region and the most probable amino acids involved in chromate-protein binding.

## Materials and Methods

### Bacterial Strains, Culture Conditions and DNA Manipulation

The bacterial strains and plasmids utilized in this study are listed in Table 1. The primers used in this work are indicated in Table S1. In general, bacteria were grown at 37°C in Luria–Bertani (LB) medium with vigorous aeration. When required, antibiotics were added at the following concentrations: 30 mg/ml kanamycin and 15 mg/ml gentamycin. Routine DNA manipulations, including PCR amplifications from genomic DNA templates, were performed as previously described [22]. Preparations of plasmid DNAs were performed using the JetQuick Mini-spin kit (Genomed, Lohne, Germany) and plasmid extraction was performed according to the manufacturer’s instructions.

**Table 1:** Bacterial strains and plasmids used in this study.

Strains and plasmids	Relevant genotype or characteristic(s)	Reference or source
***O. tritici*** ** 5bvl1**	Cr(VI)^r^	17
***E. coli*** ** DH5α**	F^−^ ω80*dlac*Δ(*lacZ*)M15 Δ (*lacZYA-argF*) U169 *endA1 recA1 hsdR17* (r_K_^−^, m_K_^+^)*deoR thi-1phoA supE44* λ^−^ *gyrA96 relA1*	Invitrogen
***E. coli*** ** BL21 (DE3)**	F^−^ *ompT hsdS* (r_B_^−^, m_B_^−^) *gal dcm lacY1*(DE3)	Novagen
**pProbe-NT**	Km^r^, promoterless plasmid, gfp	23
**pET30a**	Km^r^, expression plasmid	Novagen
**pchrGFP1**	pProbe-NT containing DNA fragment including *chr*promoter region between positions −170 and +1	This study
**pchrGFP2**	pProbe-NT containing DNA fragment including *chr*promoter region between positions −90 and +96	This study
**pchrGFP3**	pProbe-NT containing DNA fragment including *chr*promoter region between positions −90 and +1	This study
**pChrBGFP**	pchrGFP1 containing the total *chrB* gene upstream of *gfp*	This study
**pChrBGFPn**	pchrGFP1 containing the partial *chrB* gene (ChrB N-terminal) upstream of *gfp*	This study
**pChrBGFPc**	pchrGFP1 containing the partial *chrB* gene (ChrB C-terminal) upstream of *gfp*	This study
**pchrGFPmutP**	pchrGFP1 containing a mutation into the imperfect inverted repeat	This study
**pChrBGFPmutP**	pChrBGFP containing a mutation into the imperfect inverted repeat	This study
**pchrGFPGm**	pchrGFP1 containing a gentamicin resistance gene	This study
**pChrBGFP-R18A**	pChrBGFP with Arg18 changed to Ala	This study
**pChrBGFP-R23A**	pChrBGFP with Arg23 changed to Ala	This study
**pChrBGFP-R175A**	pChrBGFP with Arg175 changed to Ala	This study
**pChrBGFP-R180A**	pChrBGFP with Arg180 changed to Ala	This study
**pChrBGFP-R182A**	pChrBGFP with Arg182 changed to Ala	This study
**pChrBGFP-R187A**	pChrBGFP with Arg187 changed to Ala	This study
**pChrBGFP-R195A**	pChrBGFP with Arg195 changed to Ala	This study
**pChrBGFP-R196A**	pChrBGFP with Arg196 changed to Ala	This study
**pChrBGFP-C213A**	pChrBGFP with Cys213 changed to Ala	This study
**pChrBGFP-H229A**	pChrBGFP with His229 changed to Ala	This study
**pChrBGFP-A241R**	pChrBGFP with Ala241 changed to Arg	This study
**pChrBGFP-G244R**	pChrBGFP with Gly244 changed to Arg	This study
**pChrBGFP-H258A**	pChrBGFP with His258 changed to Ala	This study
**petChrBHis6**	pET30a for overproduction of ChrB with an C-terminal hexahistidine tag	This study
**petChrB**	pET30a for overproduction of ChrB without any tag	This study
**petChrB-R18A**	petChrBHis6 with Arg18 changed to Ala	This study
**petChrB-R23A**	petChrBHis6 with Arg23 changed to Ala	This study
**petChrB-R180A**	petChrBHis6 with Arg180 changed to Ala	This study
**petChrB-R187A**	petChrBHis6 with Arg187 changed to Ala	This study
**petChrB-H229A**	petChrBHis6 with Arg229 changed to Ala	This study
**petChrB-H258A**	petChrBHis6 with Arg258 changed to Ala	This study

### Identification of the Promoter Region by Construction of Reporter Gene Plasmids

**Figure 1:**
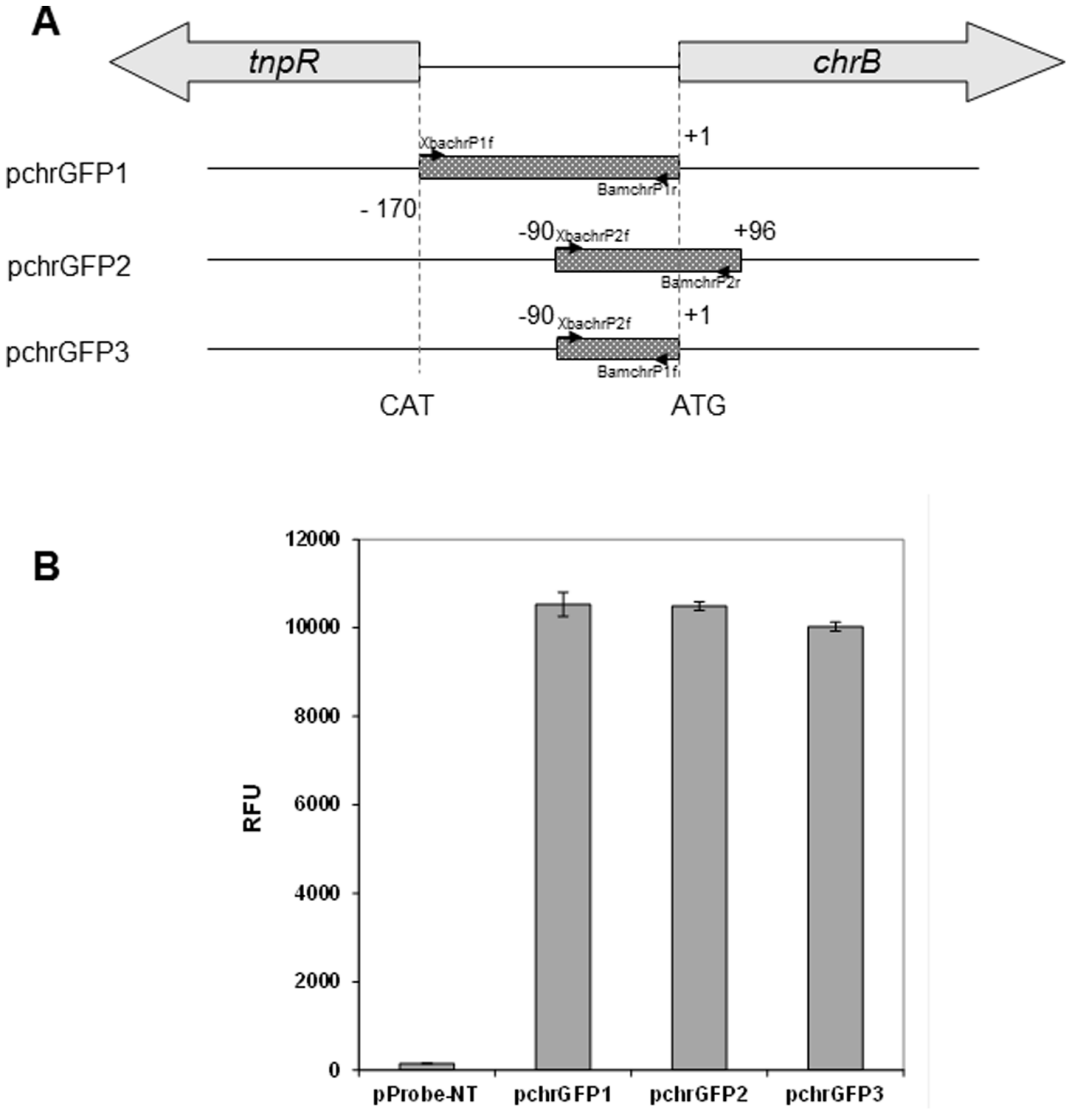
Localization of the *chr* promoter within the *tnpR-chrB* intergenetic region using *gfp* transcriptional fusions. A) PCR fragments containing the promoter portions (indicated by the boxes) were cloned upstream of a promoterless *gfp* on pProbe-NT. Sequences are numbered relative to the first nucleotide of the *chrB* start codon. Primer pairs used in this work are indicated. B) Green fluorescence of *E. coli* cells harboring the different constructs. The values represent averages and standard deviations of three replicates.

Several DNA sequences shown in Fig. 1 were amplified with specific primer pairs. The PCR-amplified DNA fragments were digested with respective restriction enzymes and ligated into cleaved vector pProbe-NT [23] yielding pchrGFP1; pchrGFP2 and pchrGFP3. The resulting plasmids, which contained the putative promoter sequences upstream of the promoterless *gfp* gene, were transferred into *E. coli* DH5α by transformation. Correct gene insertion was confirmed by DNA sequencing, performed by Macrogen (Macrogen Europe Netherlands). The promoter activities were determined by measuring the green fluorescence using a fluorimeter.

### Cloning and Purification of ChrB-His6

The *chrB* coding sequence was amplified from *O. tritici* 5bvl1 DNA using specific primers engineered to contain restriction sites for *Nde*I and *Sal*I (NdechrB1f and SalchrB1r). The stop codon was removed from the reverse primer to allow the translation of a C-terminal His6-tag encoded by the expression vector pET30a (Novagen, San Diego, CA). The amplified product was digested with the indicated restriction enzymes and cloned into pET30a generating petChrBHis6, which C-terminus recombinant ChrB was fused to a His6-tag. For protein expression, the plasmid was used to transform *E. coli* BL21 (DE3) cells and the construct was verified by DNA sequencing. To overexpress and purify ChrB-His6, *E*. *coli* BL21 (DE3) carrying plasmid petChrBHis6 was grown overnight at 37°C in LB containing kanamycin. The culture was diluted 1∶10 into 1L of LB with kanamycin and incubated at 37°C until it reached an optical density at 600 nm of 0.5. Then, isopropyl-D-thiogalactopyranoside (IPTG) was added to a final concentration of 0.5 mM, and incubation was continued overnight at 25°C.

Bacterial cells were harvested, resuspended in 20 mM sodium phosphate buffer at pH 7.4 with 0.5 M NaCl and 20 mM imidazole. A protease inhibitor cocktail (Roche, Mannheim, Germany), 10 µg/ml DNAse I and 5 mM MgCl_2_ were added to the suspension. Cells were disrupted twice in a French-press cell followed by centrifugation (15000×g, 4°C, 40 min). The recombinant ChrB-His6 protein was purified in a prepacked Ni-Sepharose high-performance column (His-Prep FF 16/10) equilibrated with 20 mM sodium phosphate, pH 7.4, 0.5 M NaCl, and 20 mM imidazole. Elution was carried out with 500 mM imidazole and the eluted fractions containing the majority of ChrB-His6 were concentrated by centrifugation in 30 kDa cutoff centricons (Millipore, Bedford, MA), equilibrated with 20 mM Tris, pH 7.4. The purity of fractions was assessed by electrophoresis on a 0.1% sodium dodecyl sulfate (SDS)-10% polyacrylamide gel, followed by Coomassie blue staining. The purified ChrB-His6 protein was stored in buffer Tris 20 mM, pH 7.4 in ice. ChrB-His6 protein concentrations were determined by using the Bradford assay (Bio-Rad, Hercules, CA) and bovine serum albumin (BSA) (Sigma, St. Louis, MO) as the protein standard.

### Cloning and Purification of ChrB

The *chrB* coding sequence was also amplified from *O. tritici* 5bvl1 DNA using specific primers engineered to contain the restriction sites for *Nde*I and *Eco*RI (NdechrB1f and EcochrB1r, respectively) but in this approach a stop codon was present into reverse primer to obtain a recombinant protein without a His-tag. The PCR product was digested with these enzymes and cloned into pET30a generating petChrB. After DNA sequencing confirmation, the plasmid was introduced into *E. coli* BL21 (DE3) cells. To overexpress ChrB (without His-tag), *E*. *coli* BL21 (DE3) carrying plasmid petChrB was grown and submitted to the same growth conditions as indicated above.

Cell extracts were loaded onto a DEAE-Sepharose column equilibrated with 20 mM Tris buffer at pH 7.5. Elution was carried out with a linear gradient of 0 to 1 M NaCl and protein fractions were visualised in SDS-PAGE as described above. The fractions with the most amount of ChrB were pooled, concentrated by centrifugation in 30 kDa cutoff centricons, desalted and loaded onto a Q-Sepharose column equilibrated with the same buffer followed by a linear gradient of 0 to 0.5 M NaCl. Desalted samples were loaded onto Resource-Q columns, equilibrated with the same buffer and eluted with linear gradients of 0 to 0.4 M NaCl. Fractions of interest were concentrated by centrifugation and equilibrated with 20 mM Tris, pH 7.4. Protein content of the samples was determined by the Bradford assay; the ChrB fraction purity was determined by SDS-PAGE and stored in ice for protein assays. The identity of the purified ChrB was confirmed by Peptide Mass Fingerprinting (IPATIMUP Proteomics Unit, Porto, Portugal).

### Chemical Crosslinking of ChrB

To determine whether ChrB is in oligomer form, chemical crosslinking assays were performed using glutaraldeyde. Reaction mixtures containing 8 µg of purified ChrB or ChrB-His6 in crosslinking buffer (20 mM NaCl, 10 mM KCl, 2 mM DTT in 20 mM Hepes, pH 7.5) were incubated with glutaraldehyde to a final concentration of 0.1%, and the reaction mixture was incubated for 2; 5, 10; 30 and 60 min at 30°C. Crosslinking was terminated by adding SDS-PAGE sample buffer, and the samples were heated at 95°C for 5 min and analyzed by 10% SDS-PAGE.

### Construction of Chromate Reporter Plasmids

The *chr* promoter and *chrB* gene were amplified from *O. tritici* 5bvl1 DNA, using the primers XbachrP1f and EcochrB1r, by standard PCR condition. The PCR-amplified DNA fragment (1109 bp) was digested with *Xba*I and *EcoR*I and ligated upstream of *gfp* gene of vector pProbe-NT [23] yielding pChrBGFP. Other constructions were also performed to evaluate reporter plasmids carrying partial *chrB* sequences. Thus, N-terminal of ChrB was obtained using XbachrP1f and EcochrB2r primers and was cloned upstream of *gfp* gene resulting pChrBGFPn. To obtain the pChrBGFPc, the ChrB C-terminal sequence, amplified by using BamChrBf and EcoChrB1r primers, was cloned into plasmid pchrGFP1. All these plasmids were introduced into *E. coli* DH5α and confirmed by DNA sequencing.

### Site-directed Mutagenesis

The pChrBGFP and petChrBHis6 plasmids were utilized as PCR templates to induce frequent nucleotide misincorporation into chromate reporter plasmid and recombinant over-expressed ChrB protein, respectively. Mutant variants of the wild-type ChrB protein were generated by overlap extension PCR according to the method of Ho and collaborators [24]. Briefly, the mutagenic reaction mixtures (50 µl) contained: 5 µl of enzyme reaction buffer, 0.2 mM of each dNTP, 150 ng of each oligonucleotide primer, 10 ng of DNA plasmid and 2.5U of Platinum Pfx polymerase (Invitrogen, Carlsbad, CA). The sequence of oligonucleotides used in mutagenesis is described in Table S1, in the supporting information. The reaction was conducted in a thermal cycler (Bio-Rad) for 16 cycles of 94°C for 1 min denaturation, 60°C for 1 min annealing, and 68°C for 16 min extension. PCR reactions were digested with 10 U of *Dpn*I for 2 hours at 37°C and then 5 µl were transformed in 100 µl of competent *E. coli* DH5α or *E. coli* BL21. Colonies were selected on kanamycin LB plates and the point mutations were confirmed by DNA sequencing analyses.

### Electrophoretic Mobility Shift Assay (EMSA)

The EMSA was conducted as described previously [25]. The promoter sequence DNA probe was generated using the primer pair XbachrP1f and BamchrP1r. The PCR product was gel purified and biotin end labelled by using LightShift Chemiluminescent EMSA kit (Pierce, Rockford, IL). In general, DNA binding assay was performed in a 20 µl reaction volume containing binding buffer (10 mM Tris pH 7.5, 50 mM KCl, 1 mM DTT), 1 µg poly(dI-dC), 10% glycerol, 1 mM MgCl_2_, 30 fmol DNA probe and different concentrations of purified ChrB. To show the effect of chromate in DNA-protein binding assays, the protein was first incubated with chromate (10, 100 or 1000 µM) for 10 min at room temperature and then DNA labelled probe was added. In competition assays, 1 to 5 µg of cold probe was used to challenge the labelled probe. After incubation at 25°C for 20 min, the samples were loaded onto a native 6% polyacrylamide gel and electrophoresed in 1×Tris-borate EDTA (TBE) buffer for 70 min at 100 mA. Following gel electrophoresis, the complexes were electroblotted to nylon membrane (Roche) and detected using a chemiluminescence based nucleic acid detection kit (Pierce).

### Bacterial Double Plasmid Assays

The previous promoter reporter plasmid pchrGFP1 have been engineered to introduce an additional antibiotic resistance cassette as reported in [26]. In this work, a plasmid pchrGFP1 carrying an additional gentamicin resistance gene was constructed and it was inserted into *E. coli* DH5α cells yielding pchrGFPGm.

Competent *E. coli* BL21 cells carrying the plasmid petChrBHis6 or the several mutated plasmids (petChrBR18A; petChrBR23A; petChrBR180A; petChrBR187A; petChrBH229A) were co-transformed with pchrGFPGm and and co-transformants were selected using LB medium that contained kanamycin (30 mg/mL), and gentamicin (15 mg/mL). The co-transformed strains were submitted to chromate and green fluorescence was evaluated.

### *E. coli* Reporter Activity Assay

The *E. coli* reporter cells were grown overnight, on a shaker (150 rpm) at 30°C in LB medium and used as inoculum. Cultures of these strains were performed in Tris-buffered mineral salts medium [27] supplemented with glucose 0.5% and vitamins [17], designated by TMM. Cells were grown at 37°C up to an OD600nm of about 0.2–0.3, corresponding to cells at exponential phase and were stressed by addition of the indicated concentration of chromate. The stock chromate solution (1 M) was prepared by dissolving the analytical grade metal salt (as K_2_CrO_4_) in ultra pure water and sterilized by filtration. The stressed cultures were then incubated at 37°C, for the period of 3 hours. During this period, reaction volumes of 200 µl were transferred onto clear 96-well plates (Thermo Labsystems, Helsinki, Finland) and GFP fluorescence intensity was measured in triplicate.

### Spectrofluorometry

Fluorescence intensity was measured with a Gemini EM Fluorescence Microplate Reader (Molecular Devices) with emission, excitation and cutoff wavelengths at 510, 480 and 595 nm, respectively. Relative fluorescence unit (RFU) is defined as the culture fluorescence relative to culture biomass at OD 600 nm SpectraMax Plus384 Absorbance Microplate Reader (Molecular Devices).

## Results

### Identification of *chr* Promoter Region by Construction of Promoter-*gfp* Fusion Plasmids

In order to identify the shortest *chr* promoter region, sequences immediately upstream of *chrB* gene and partial genetic fragments of *chrB* of Tn*OtChr* of *O. tritici* 5bvl1 were analysed. The subfragments indicated in Fig. 1A, created by PCR amplification were introduced upstream of *gfp* gene to construct the plasmids pchrGFP1, pchrGFP2 and pchrGFP3. These plasmids and pProbe-NT (negative control) were transformed into *E. coli* cells, respectively. No green fluorescence was detected with control sample, whereas high GFP activity was detected in cells carrying the other plasmids (Fig. 1B). Similar green fluorescence was obtained when these cells were incubated with different concentrations of chromate (data not shown). These data suggest that *chr* promoter sequence might be localised inside the last fragment, therefore between bp –90 and +1 with respect to the *chrB* translational start nucleotide. The high fluorescence signals achieved either in absence or presence of chromate indicate that this *chr* promoter acted as the constitutive promoter in the absence of the *chrB* gene.

### Identification of Putative Protein Binding Site in *chr* Promoter through Mutagenesis

A DNA fragment containing the *chr* promoter and *chrB* gene was also amplified and cloned upstream of *gfp* gene resulting in pChrBGFP. *E. coli* cells were then transformed with this plasmid and showed fluorescence, only when recombinant cells were submitted to chromate ions. This finding indicates that ChrB works as a regulatory protein and might bind to DNA.

**Figure 2:**
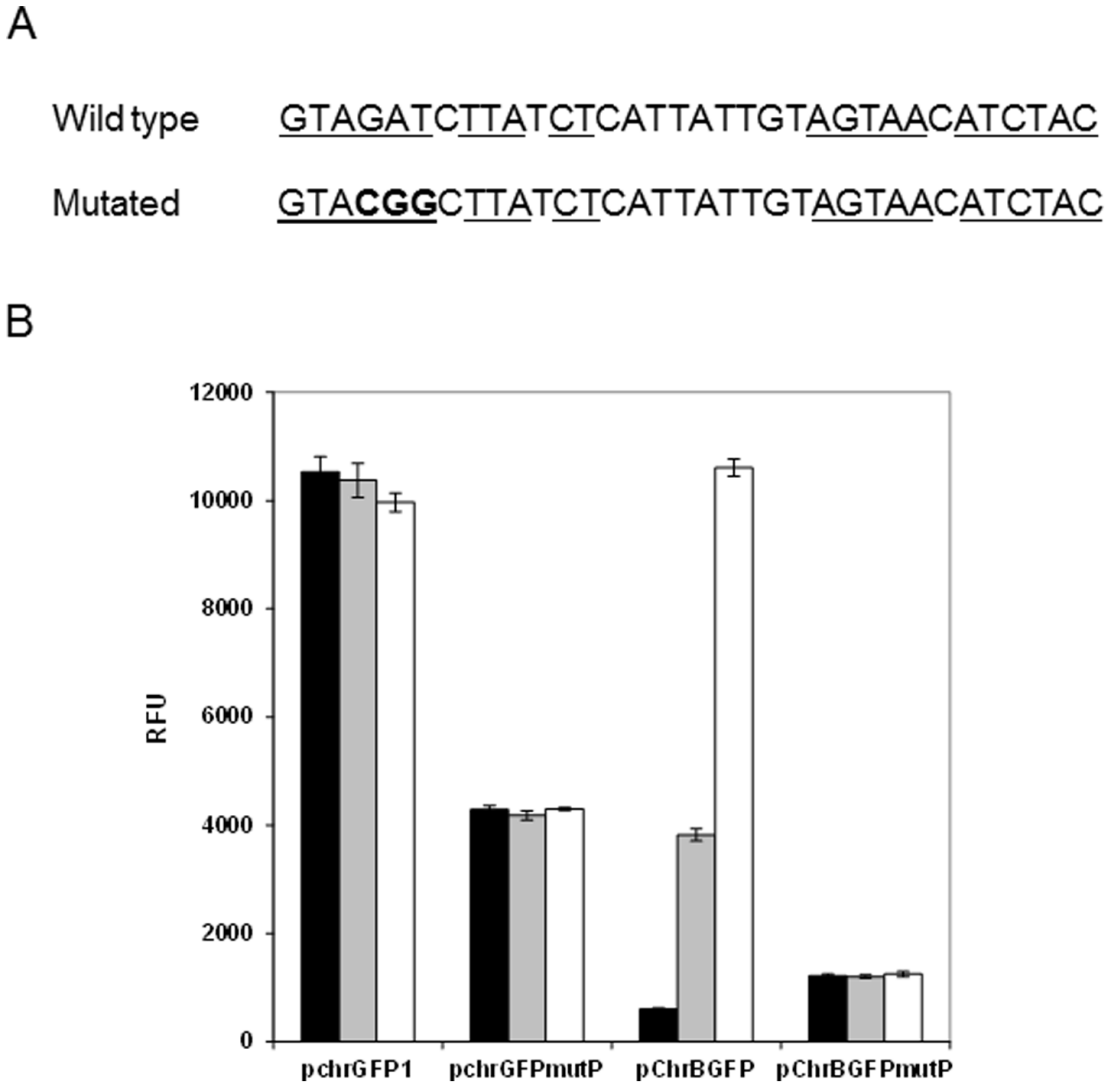
Mutagenesis analysis of the putative protein binding site within *chr* promoter region. A) Imperfect inverted repeat of the original and mutated sequence. The mutated nucleotides are shown in bold. B) Fluorescence of *E. coli* carrying the mutated and no-mutated plasmid constructs. Fluorescence was measured after 3 h of growth in medium without chromate (black bars), with 1 µM (grey bars) and 10 µM (white bars) of chromate. The values represent averages and standard deviations of three replicates.

A close examination of the previously identified *chr* promoter revealed the presence of a 12 bp imperfect inverted repeat separated by a nine base pair linker (GTAGAT CTTATCTCATTATTGTAGTAACATCTAC ) localised between bp −37 and −4 with respect to the initial ATG of *chrB* gene. The relevance of the inverted repeat was tested by nucleotide mutagenesis. Three nucleotides of the motif in constructs pchrGFP1 and pChrBGFP were exchanged resulting pchrGFPmutP and pChrBGFPmutP, respectively (Fig. 2A). Comparing the fluorescence results from mutants with the original recombinant strains, this mutation affected the GFP production. Continuous green fluorescence or chromate-induced fluorescence was detected by pchrGFP1 or pChrBGFP cells respectively, however, respective mutant cells revealed low fluorescence signals either in presence or in absence of chromate (Fig. 2B). These results indicate that the predicted DNA motif is relevant for protein regulation. In order to further confirm the importance of this promoter motif, EMSA experiments using the mutated promoter probe were conducted (data not shown). These assays showed that ChrB is still able to bind to the mutated promoter. Therefore, together, the results seem to indicate that ChrB is able to bind the mutated promoter but does not function as a regulator under these conditions.

### ChrB is not Functional as Partial Protein

**Figure 3:**
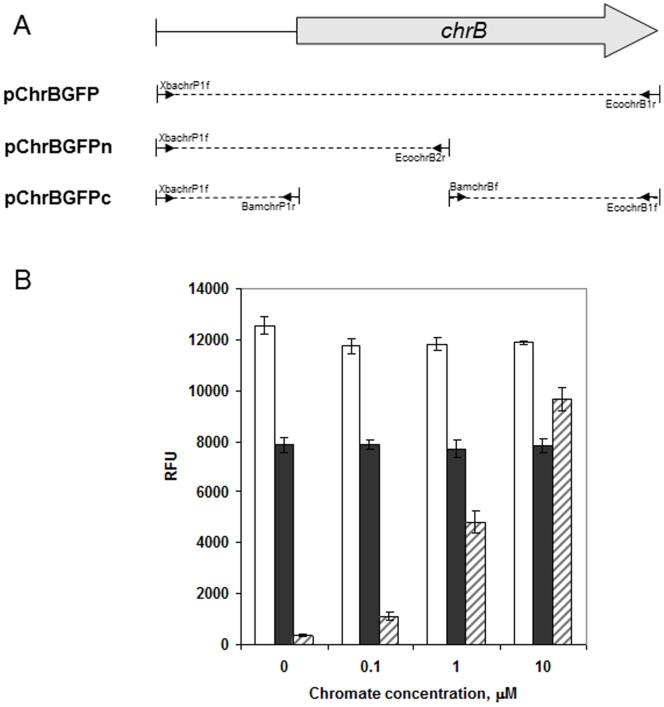
Study of the functionality of ChrB (total or partial) using reporter plasmids. A) Total or partial *chrB* (coding for N-terminal or C-terminal ChrB), with their promoter sequence, were cloned upstream of *gfp* gene in pProbe-NT (Table1). Primer pairs used in this work are indicated. B) Fluorescence of *E. coli* carrying the different plasmids constructs; pChrBGFPn (white bars), pChrBGFPc (grey bars) and pChrBGFP (dashed bars). Fluorescence was measured after 3 h of growth in medium without chromate and with increasing chromate concentrations. The values represent averages and standard deviations of three replicates.

The effect of the deletion of N-terminal or C-terminal of ChrB was examined for their ability to regulate the green fluorescence expression. When partial genes, with their promoter sequence, were cloned upstream of *gfp* gene, no regulation of fluorescence emission was observed (Fig. 3). In these experiments, *E. coli* cells were able to express continuously GFP protein, which did not changed by absence or presence of chromate. These results suggest that amino-terminal and carboxyl-terminal are required for functionality of ChrB.

### Overexpression and Purification of ChrB and ChrB-His6

**Figure 4:**
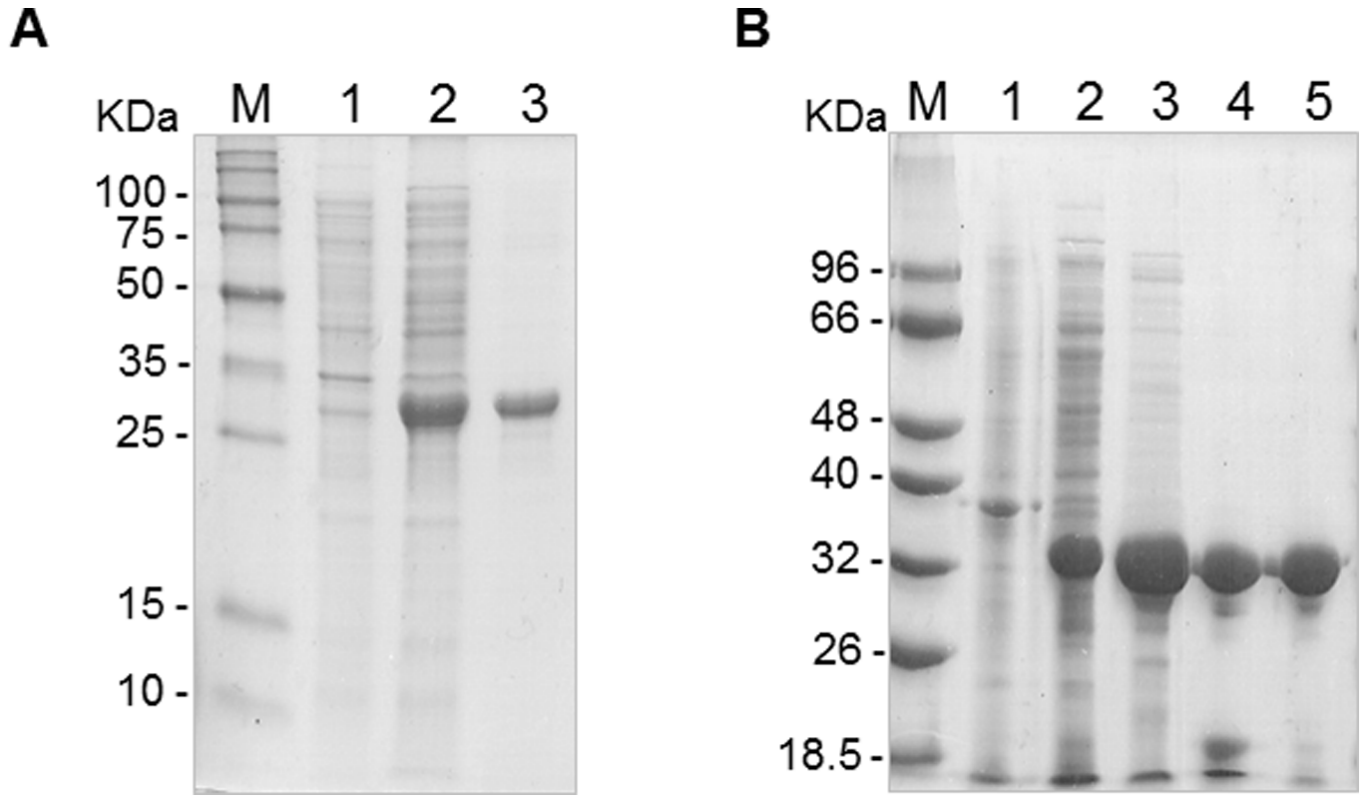
Purification of the ChrB protein. A) ChrB was overexpressed with a carboxy-terminal 6-histidine tag in *E. coli* BL21 (DE3). Lane 1, whole-cell extract from uninduced cells containing pET30a expressing ChrB-His6; lane 2, whole-cell extract from induced cells containing pET30a expressing ChrB-His6; lane 3, purified ChrB-His6 by Ni^2+^ nitrilotriacetic acid affinity chromatography. B) ChrB was overexpressed without any tag in *E. coli* BL21 (DE3). Lane 1, whole-cell extract from uninduced cells containing pET30a expressing ChrB; lane 2, whole-cell extract from induced cells containing pET30a expressing ChrB; lane 3, purified ChrB by DEAE chromatography; lane 4, purified ChrB by Sepharose chromatography; lane 5, purified ChrB by resource chromatography.

In order to obtain the ChrB protein, cloning, gene expression and protein purification experiments were conducted, which resulted in production of large amounts of protein in the soluble form. The protein ChrB-His6 was overexpressed in *E. coli* and purified by Ni^2+^ affinity chromatography. The apparent molecular mass of purified protein was 35 KDa, which is close to 35.8 KDa calculated for the 312 amino acid protein (Fig. 4A). Moreover, a second strategy of overexpression and purification of ChrB was used to ensure that His-tag did not affect the behaviour or conformation of protein. Thus, the *chrB* gene including the stop codon was cloned into plasmid pET30a. Figure 4B shows the progression of the purification on SDS-PAGE, where is on evidence a single band corresponding to a molecular mass of 35 kDa, corresponding to ChrB protein. The identity of this protein was determined by Peptide Mass Fingerprinting and corresponded to the predicted sequence of ChrB.

### ChrB Exists as Oligomers

**Figure 5:**
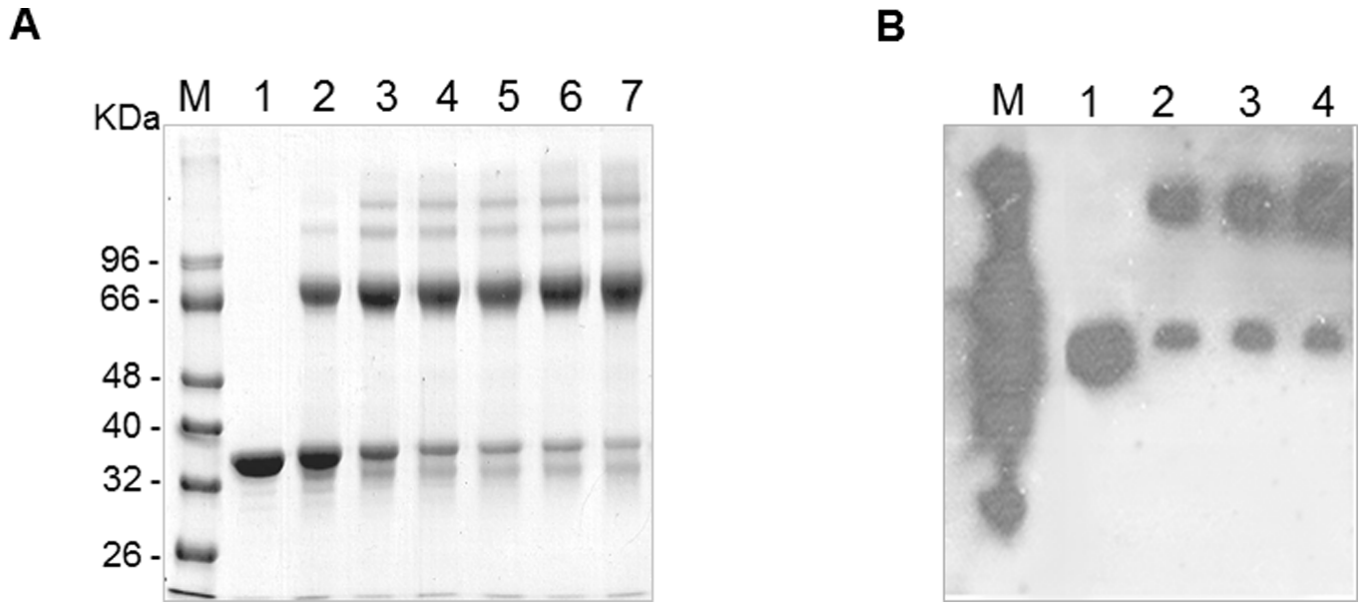
Chemical crosslinking assays. A) SDS PAGE of purified ChrB in glutaraldehyde crosslinking assays: lane 1, ChrB; lane 2, ChrB+glutaraldehyde (2 min); lane 3, ChrB+glutaraldehyde (5 min); lane 4, ChrB+glutaraldehyde (10 min); lane 5, ChrB+glutaraldehyde (15 min); lane 6, ChrB+glutaraldehyde (30 min); lane 7, ChrB+glutaraldehyde (1 hour). B) Immunoblot of purified ChrB-His6 in glutaraldehyde crosslinking assays: lane 1, ChrB-His6; lane 2, ChrB-His6+ glutaraldehyde; lane 3, ChrB-His6+ target *chr* promoter DNA+glutaraldehyde; lane 4, ChrB-His6+ glutaraldehyde+Cr(VI).

To determine whether ChrB existed as monomer or in oligomeric status, chemical crosslinking experiments were performed on purified ChrB-His6 using glutaraldehyde. Analysis of reaction mixtures that contained purified ChrB-His6 with glutaraldehyde resulted in shifting position bands on SDS-PAGE. Figure 5A shows that increasing incubation time of the protein with glutaraldehyde caused the appearance of a strong band on the gel, corresponding to the position of a dimer (approximately 70 KDa) and weak bands with higher molecular weights. These results indicated that ChrB forms oligomers and exists predominantly as dimers. Similar results were obtained when ChrB without His-tag was used (results not shown). Considering that ChrB might interact with DNA and Cr(VI), cross-linking experiments of the protein with DNA promoter sequence and/or Cr(VI) were performed and the reaction products were analysed by using immunoblots probed with anti-His antibody (Fig. 5B). Addition of chromate and/or target DNA sequence had no effect on multidimerization.

### ChrB Binds to the Promoter Region of *chr* Operon

**Figure 6:**
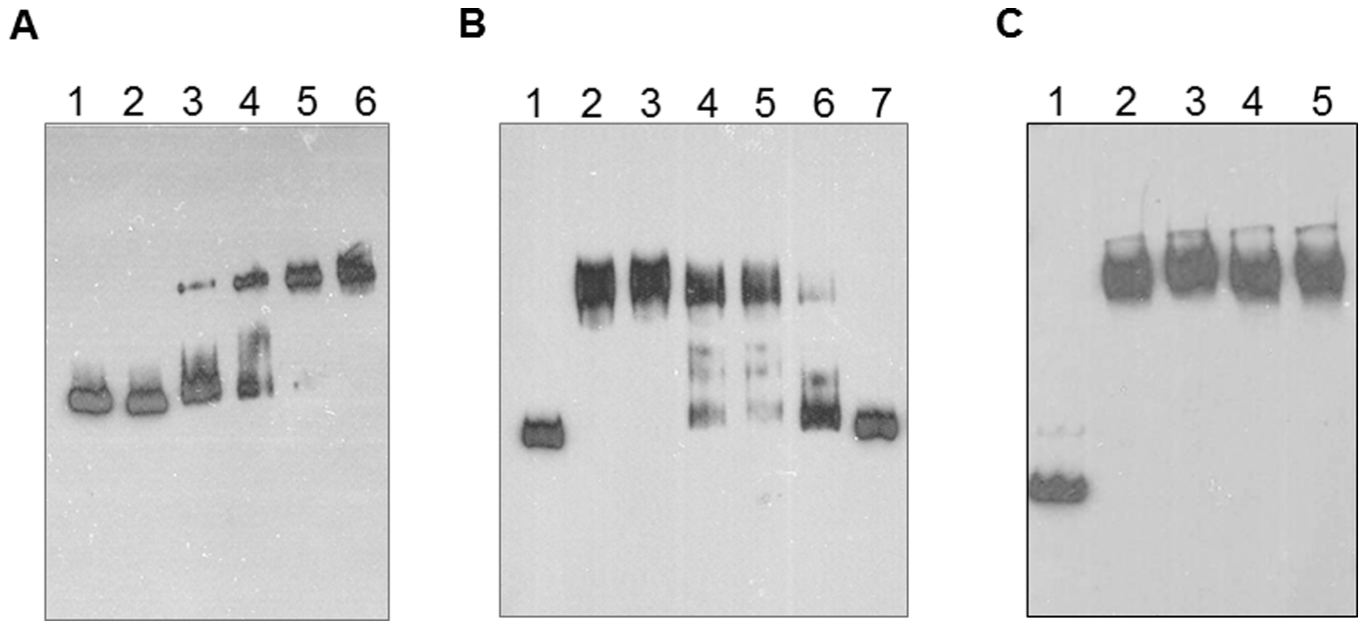
Gel mobility shift assays. A) Band shift assays were performed by incubating the DNA fragment (*chr* promoter) with increasing concentration of ChrB protein. Lane 1, competitor protein (EBNA extract from Pierce); lane 2, 0 µM ChrB; lane 3, 1 µM ChrB; lane 4, 3 µM ChrB; lane 5, 10 µM ChrB; lane 6, 30 µM ChrB. B) DNA fragment was incubated with ChrB (10 µM) in the absence and in the presence of unlabeled competitor DNA. Lane 1, without protein; lane 2; 0 µg/µl; lane 3, 1 µg/µl; lane 4, 10 µg/µl; lane 5, 50 µg/µl; lane 6, 100 µg/µl; lane 7, 250 µg/µl of competitor DNA. C) EMSA assays with or without chromate. The *chr* promoter was incubated with ChrB protein (10 µM) and with increasing concentrations of chromate. Lane 1, without protein (control); lane 2, without Cr(VI); lane 3, with 10 µM Cr(VI); lane 4, with 100 µM Cr(VI); lane 5, with 1 mM Cr(VI).

To demonstrate that ChrB binds to the *tnpR-chrB* intergenic region, electrophoretic mobility shift assays were performed, which clearly demonstrated that ChrB bound to the promoter probe DNA and retarded its migration in a concentration-dependent fashion (Fig. 6A). The shifted band became visible at ChrB concentrations of 1 µM and higher concentrations of protein increased the amount of shifted labelled DNA probe. Labelled DNA was challenged with several-fold excess of non-labelled probe DNA to determine the specificity of binding of ChrB to the DNA probe. Prebound ChrB exchanged with non-labelled DNA probe in the presence of a large excess of poly dI-dC (Fig. 6B) demonstrating that ChrB binds specifically to the *chr* promoter region. Moreover, ChrB did not switch of from DNA independently of the chromate concentrations tested (Fig. 6C).

### Design and Construction of ChrB Mutants

**Figure 7:**
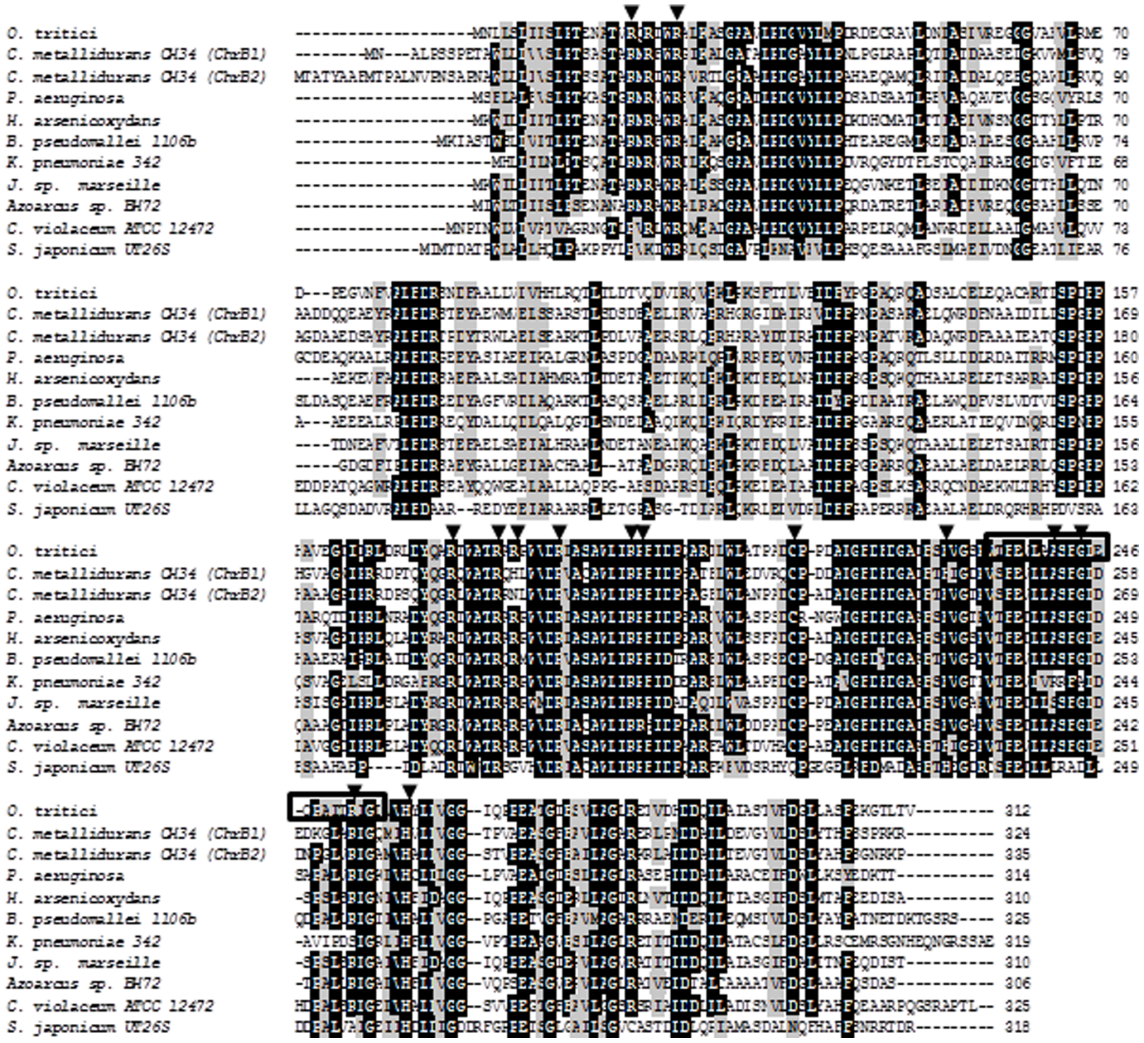
Alignment of ChrB homologues from different organisms. Amino acid sequences (obtained from NCBI) were aligned via CLUSTAL W. *Ochrobactrum tritici* 5bvl1 (ABO70326), *Pseudomonas aeruginosa* (AEQ93502); *Cupriavidus metallidurans* CH34, ChrB1 and ChrB2 (ABF13062 and ABF10734, respectively); *Herminiimonas arsenicoxydans* (CAL61077); *Burkholderia pseudomallei* 1106b (EES21856); *Klebsiella pneumoniae* 342 (YP_002238121); *Janthinobacterium* sp. Marseille (ABR91486); *Azoarcus* sp. BH72 (CAL95580); *Chromobacterium violaceum* ATCC 12472 (AAQ58595); *Sphingobium japonicum* UT26S (BAI96957). Highly conserved residues in ChrB homologues (black shading), similar residues in ChrB homologues (grey shading), conserved residues chosen for mutagenesis approach (arrow) and the putative HTH motif (into the box).

Since no crystal structure of the ChrB protein or close relatives have been determined, it was not possible to either predict rigorously the tertiary structure of ChrB or obtain a model for effector interaction with this protein. Despite of these difficulties, which impact the prediction of which amino acids in the ChrB could be implied in chromate interaction, the amino acid sequence of ChrB from *O. tritici* was aligned with a large number of available ChrB sequences. Based on these alignments, a region with a high homology was visible, rich in residues of arginine (Fig. 7). Some of the most conserved basic residues, namely arginines and histidines, were chosen to be mutated in order to identify the most probable amino acids that interact with chromate. A group of mutations were created to validate or refute the hypothesis (Fig. 7). Mutations in this group of basic residues were designed to alter the chemical nature of the residue (charged to non-charged hydrophobic or to non-charged hydrophilic). Therefore, the arginines (R175; R180; R182; R187; R195; R196) and histidines (H229; H258) selected were changed to alanines and glycines. Additionally, one conserved residue of cysteine (C213) was also chosen for mutation.

**Figure 8:**
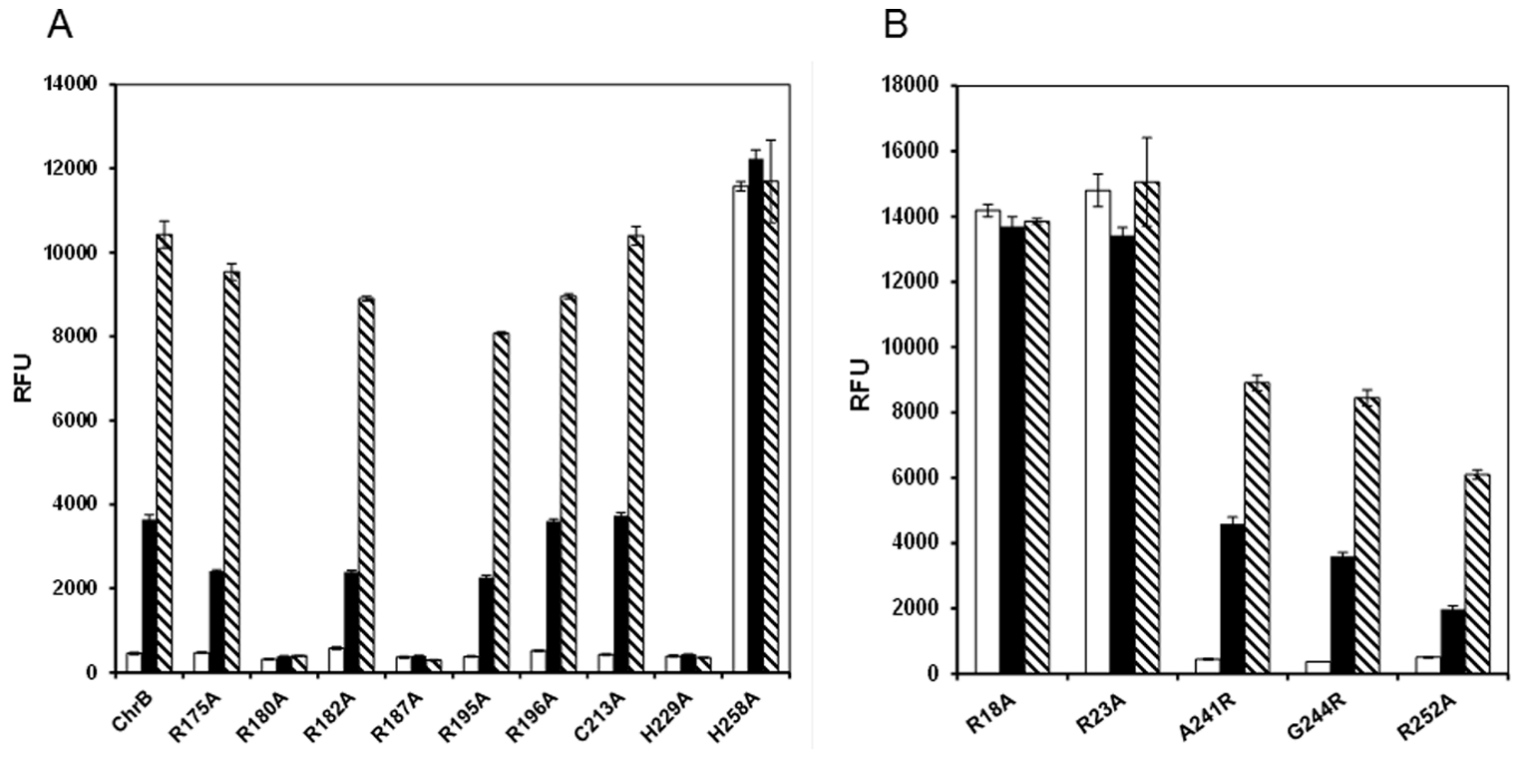
GFP expression of pChrBGFP and mutants. A) Green fluorescence of pChrBGFP (ChrB no mutated) and pChrBGFP mutants, in which, ChrB is mutated in R175A; R180A; R182A; R187A; R195A; R196A; C213A, H229A and R258A. B) Green fluorescence of pChrBGFP mutants, in which, ChrB is mutated in R18A; R23A; A241R; G244R and R252A. Fluorescence was measured after 3 h of growth in medium without chromate (white bars), with 1 µM (black bars) and 10 µM (dashed bars) of chromate. The values represent averages and standard deviations of three replicates.

All mutants were tested in *E. coli* for GFP expression during exponential growth in the presence or absence of chromate. Fig. 8 gives representative GFP induction values after 3 h induction time compared to those of the strain carrying wild type ChrB. The group of mutations which changed the positively charged conserved residues and the conserved cysteine produced the following results (Fig. 8A): 1) mutations R180A, R187A and H229A completely abolished GFP expression upon chromate addition; 2) mutations R175A, R182A, R195A, R196A and C213A did not significantly affect chromate dependent GFP expression; 3) changing His258 to Ala resulted in a very high GFP expression in the absence of chromate when compared to wild-type ChrB activation in *E. coli*. For all of these residues, the Arg, His and Cys substitutions to Gly were created and produced the same effect as indicated for their substitutions to Ala (data not shown). These results suggested that several residues in this conserved area, namely R180, R187 and H229, might affect interaction between chromate and protein.

ChrB is a protein able to bind DNA and since there are no studies indicating which domain is implicated in this assignment, we used two available software tools for identification of the most putative DNA-protein binding domain of ChrB. First, by using the program Network Protein Sequence Analysis [28] for detection of the helix-turn-helix (HTH) domains, a HTH motif was identified with approximately 25% of probability. This motif was emphasized on ChrB alignments shown in Fig. 7. Second, by using the program BindN, for prediction of DNA and RNA binding residues in proteins [29] the residues Arg18 and Arg23 were identified as the residues with the highest score to bind DNA with a confidence coefficient of 0.98. In order to determine whether these identified sites were relevant to the DNA binding capacity, two groups of mutations were performed via site-directed mutagenesis. The reporter activities of strains containing mutated ChrB were then assayed. The mutated strains on ChrB residues A241R, G244A, and R252A, included in the predictable HTH motif, exhibited GFP activities when exposed to increasing concentrations of chromate (Fig. 8B). These results might indicate that the selected residues, and most probably this putative motif, are not involved in ChrB-DNA interaction. On the other hand, mutation of the residues R18A or R23A seems to have changed the ability of protein to bind to the DNA. These two mutated ChrB strains expressed the GFP by a constitutive mode and independently of the absence or presence of the inducer (Fig. 8B). The results suggest that these two residues close to the N-terminus of ChrB are implicated in the binding of the protein to the DNA operator, without excluding the involvement of other residues. To assure that the amino acid modifications had not drastically changed or compromised the protein structure, mutant ChrB proteins were purified and their migration on native polyacrylamide gel and multimerization through chemical crosslinking assays were compared with non-mutated ChrB protein. Mutant ChrB proteins and wild-type ChrB (all tagged with His6) were purified by Ni-NTA chromatography. Identical protein expression, gel migration and oligomer production were obtained except for H258A mutant. In the latter, no soluble protein was detected, suggesting a drastic configuration change induced by the mutation, which led to the production of the protein in inclusion bodies.

Therefore, the former results may indicate that mutations, with exception of H258A, did not cause a significant overall change in protein configuration. Consequently, the effects on chromate dependence GFP expression were the end result of a change in a critical effector binding region or residue. Likewise, the unregulated reporter activities, exhibited on the previous results, were the result of essential residues modification on protein-DNA binding domain.

### Confirmation of ChrB Regulation by Double Plasmid Expression Analysis

**Figure 9:**
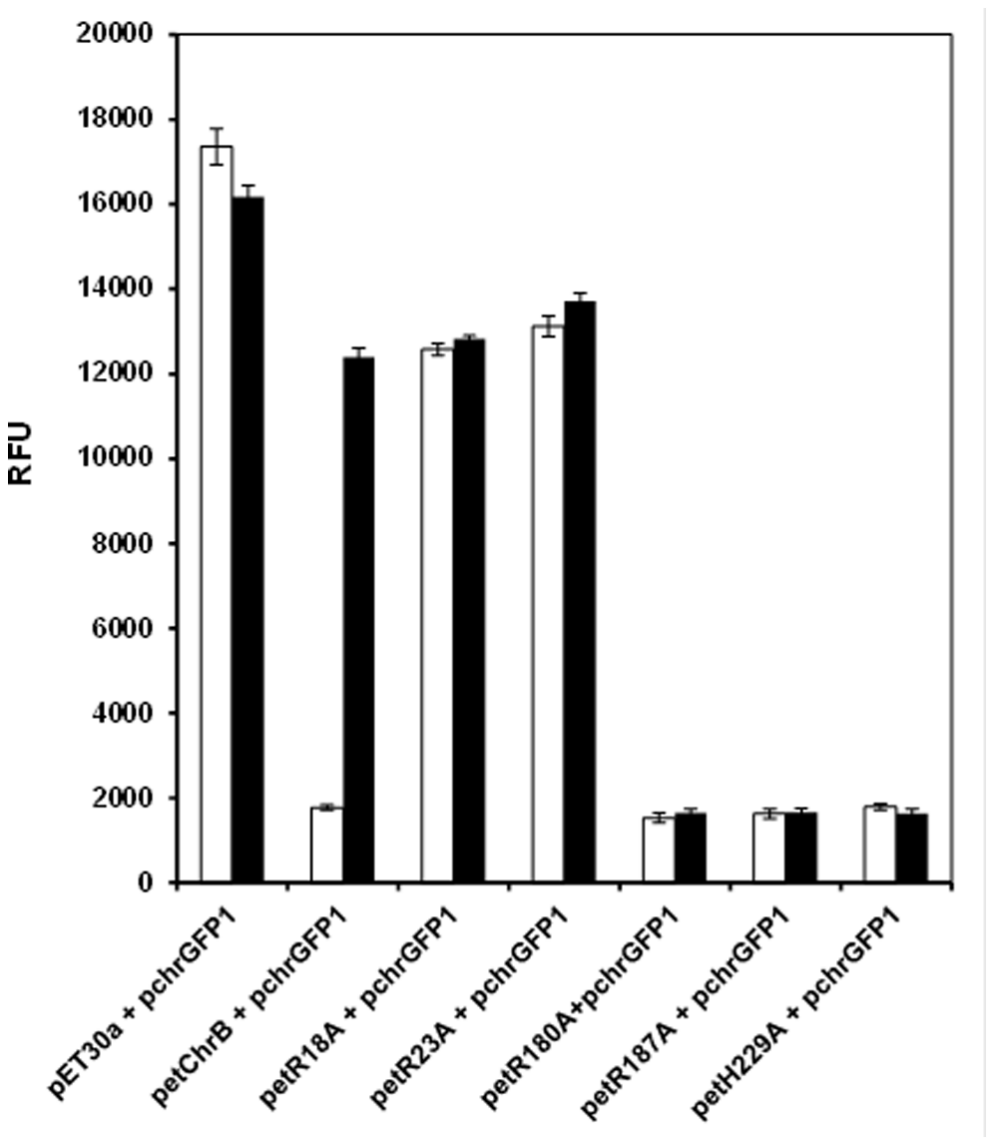
Fluorescence of *E. coli* BL21 co-transformed with two different plasmids (indicated in graphic). Fluorescence was measured after 3(white bars) and with 10 µM (black bars) of chromate. The values represent averages and standard deviations of three replicates.

To confirm the ChrB regulation of *chr* operon, a plasmid *chr/gfp* transcriptional fusion was produced, introduced into *E. coli* BL21 harboring petChrB, petChrBR18A; petChrBR23A; petChrBR180A; petChrBR187A or petChrBH229A, and the GFP activity was subsequently measured (Fig. 9). The resulting strains and the control strains carrying the empty expression vector pET30a were cultivated in minimal medium with or without the addition of chromate. When *E. coli,* complemented with a plasmid expressing wild-type ChrB was cultivated in medium with chromate, the strain exhibited green fluorescence dependent on chromate dose. In the absence of ChrB (control strain), GFP expression was high and not dependent on chromate dose, indicating that ChrB exerted a negative regulatory effect. Strains co-transformed with pchrGFP1 and petChrBR18A or petChrBR23A also exhibited high fluorescence signals even in absence of chromate. These data strengthen the previous idea that both arginines (R18 and R23) should be involved in the protein capacity to bind DNA. None of the additional strains co-transformed with pchrGFP1 and the other three petChrB mutants (petChrBR180A, petChrBR187A or petChrBH229A) exhibited GFP activity when incubated with chromate. Thus, these ChrB mutants did not allow transcription of the reporter gene under these conditions, which supported the previous data that these mutated proteins are affected at the chromate interaction site. The combination of presented results confirmed that ChrB regulates the *chr* operon expression in a strictly chromate-dependent manner.

## Discussion

Bacteria must sense and adapt to environmental concentrations of a diversity of metals in order to survive, either low concentrations of essential metals needed as micronutrient or the presence of toxic heavy metal levels. This implies that bacteria must be capable of obtaining or scavenging trace metal ions from their environment to meet basic cellular needs and, on the other hand, must have inducible resistance mechanisms when concentrations exceed physiological needs. This bacterial capacity should require multiple families of metal-responsive transcription factors that discriminate between elements and thereby activate expression of genes responsible for suitable responses. Seven main bacterial families of metal-sensing transcriptional regulators have so far been identified: ArsR–SmtB, MerR, CsoR–RcnR, CopY, DtxR, Fur and NikR. They span the detection of many transition elements such as Mn, Fe, Co, Ni, Cu, Zn, Ag/Au, Cd/Hg, as well as the p-block elements Pb and As/Sb [30]. These metal-sensor proteins are usually described as specialized allosteric proteins that regulate the transcription of genes linked to transition metal homeostasis, as a result of direct binding of a single metal ion or closely related metal ions [31]. In addition, there are some other metal-sensors belonging to structurally distinct families of regulators, that also include members not involved in metal sensing (e.g. TetR and LysR families) [32].

In contrast to the majority of the metals, only a very small number of transcription factors have been considered for chromate sensing. So far, *chrB* and *chrF* of *C. metallidurans* [20], *chrI* of *Bacillus cereus* [15] and *chrB* of *O. tritici* 5bvl1 [17] have been reported as the genes which most probably encode for regulatory proteins that control *chr* operon expression. Some roles for ChrB have been anticipated, including the activity of chromate reductase [16]. However, besides the initial speculation that ChrB acts as a regulator of gene expression, little effort has been made to assign a function to ChrB or to indicate possible mechanisms of action. Therefore, ChrB has remained as an uncharacterized protein. To fill this gap, we used a combined bioinformatics and functional approach to identify and characterize this novel chromate induced transcriptional regulator – ChrB. In contrast to the ArsR and MerR families, whose representative members have evolved to detect a far wider range of metal ions [26], the chromate reporter has been demonstrated to be very specific to this heavy metal [30].

The NCBI database includes ChrB of *O. tritici* 5bvl1 within the export chromate resistance protein group, and BLAST analyses showed in ChrB a conserved rhodanese domain at the C-terminus. Distinct proteins containing rhodanese-like modules are widely distributed among diverse proteins, and enable the identification of many subfamilies [33]. Each subfamily harbors conserved motifs in the amino acid sequence of the putative active-site loop. The rhodanese-like domain of ChrB bears weak but significant sequence similarity to the six amino acid active loop [CG(S/T)GVT] of the described 3-mercaptopyruvate sulfurtransferase (MST) proteins. Rhodanese-like domains were also found within regulatory proteins such as the arsenic resistance operon repressor ArsR. The wide variety of amino acids of the putative active-site loop of the rhodanese homology domains has been associated to different substrate specificity. Therefore, the several rhodanese protein subfamilies could be involved in different biological functions [34]. Additionally, the ChrB C-terminal domain comprises the most conserved residues and, among these, the presence of a large number of very conserved arginines was particularly intriguing. This amino acid, as well as other positively-charged residues, has been referred as possibly involved in chromate binding and recognition [15]. Since cysteines are often involved in metal-regulatory protein interactions, a conserved residue of cysteine was also evaluated in this work as a possible key regulatory residue [35], [36]. Contrary to what expected, Cys213 cannot function as a site for chromate-binding, but, on the other hand, fluorescence assays revealed that substitution of two arginines (Arg180, Arg187) and one histidine (His229) influence ChrB responsiveness to chromate. The mutagenesis strategy was also used to modify the N-terminal half protein, leading us to form the hypothesis that ChrB could exhibit two functional domains with different roles: the N-terminal being responsible for DNA-binding and the C-terminal for carrying the metal detection motives.

Our experiments revealed that ChrB works as an oligomeric form, mainly as a dimer. Characteristically, the regulatory proteins adopt oligomeric forms, and the dimeric assembly state is the most usual feature among the metal-responsive regulatory proteins. This regulation should also affect the expression of *chr* operon downstream genes, namely the *chrA*. This hypothesis is sustained by the fact that coding sequences of genes *chrB* and *chrA* are not separated but overlap in *O. tritici* genome. Transport actions are usually regulated for metalloids and metals such as arsenite and several cations (revised on [35], [37]) and therefore so is the export of chromium from cells by the chromate efflux pump, ChrA [38].

The predicted binding site of ChrB to its own promoter DNA is very close to the coding sequence. It suggests that the complex formed by ChrB and the promoter DNA inhibits the binding or sliding of RNA polymerase, causing auto-repression. Another possibility, since the addition of chromate in EMSAs assays did not lead to turning off ChrB-*chr* promoter DNA, is the complex ChrB-chromate ions glides along the DNA promoter, which may be conducive to initiation or promotion of transcription. This kind of effects of transcriptional regulators was also observed in other regulators [39].

Some evidences showed that ChrB is a chromate responsive DNA-binding regulator of transcription of the *chr* operator/promoter: (i) Purified ChrB forms complexes with the *chr* operator/promoter in vitro as shown in EMSA assays; (ii) in absence of chromate, expression of GFP activity from the *chr* operator/promoter is much more elevated in cells devoid of *chrB* gene compared with cells containing ChrB; (iii) in cells carrying *chr* operator/promoter and *chrB*, expression of fluorescence is elevated in response to chromate concentrations; (iv) mutations of specific residues of ChrB affect GFP performance.

## Conclusions

This study increases the knowledge on the cell metal homeostasis regulation mechanisms by studying the protein ChrB, which plays a significant role in chromate resistance in strain *O. tritici* 5bvl1. This research is an important first step in the characterization of potential regulatory elements of the *chr* operon, which includes the operator/promoter region and the essential amino acids that control the *chr* expression system. Further work focused on the structure of ChrB will demonstrate the value of these predicted elements in the context of chromate sensing and chromate resistance mechanisms. This study confirms that ChrB is a regulatory protein and brings new light on protein-DNA and protein-Cr(VI) interactions.

## Supporting Information

Table S1 **Oligonucleotide sequences.**
(DOC)
