# Supplementary material for: Identification and Characterization of the Transcriptional Regulator ChrB in the Chromate Resistance Determinant of Ochrobactrum tritici 5bvl1
Source: PLoS One. 2013 Nov 4;8(11):e77987. doi: 10.1371/journal.pone.0077987 (PMC3817168; doi:10.1371/journal.pone.0077987)
Supplement: Table S1 — Oligonucleotide sequences. (DOC) [file pone.0077987.s001.doc]

| **Name** | **Sequence** |
| --- | --- |
| **XbachrP1f** | CGTCTAGAGATTGCTTATTCCTATTGCCA |
| **BamchrP1r** | GTGGATCCGTCGTAGATGTTACTACAATA |
| **XbachrP2f** | CGCTCTAGATTGCGCAAATGTCCGTTTTTGCAA |
| **BamchrP2r** | GTGGATCCACCGCAGCGCCGGAGGCTTTG |
| **EcochrB1r** | CGGAATTCTCATACGGTGAGGGTCCCTTT |
| **EcochrB2r** | CGAGAATTCTCAGGCCTGGTAATCCAAACGATC |
| **NdechrB1f** | CGACATATGAATTTGCTTTCGCTTATCC |
| **SalchrB1r** | CGAGTCGACTACGGTGAGGGTCCCTTTTTC |
| **NdechrB2f** | CGACATATGACCTGGGCCACTCGCGCACGT |
| **BamchrBf** | GTGGATCCATGACCTGGGCCACTCGCGCA |
| **MutPchrf** | GCAATCTACTCAAGACTTTATTTTCTACGGCTTATCTCATTATTGTAGTAACATCTAC |
| **MutPchrr** | GTAGATGTTACTACAATAATGAGATAAGCCGTACAAAATAAAGTCTTGAGTAGATTGC |
| **chrBR18f** | CTGAAAACGCGACCGTCGCCCAACGGACGTGGCGTGC |
| **chrBR18r** | GCACGCCACGTCCGTTGCGCGACGGTCGCGTTTTCAG |
| **chrBR23f** | GTCCGGCAACGGACGTGGGCTGCCCTCAAAGCCTCCGGC |
| **chrBR23r** | GCCGGAGGCTTTGAGGGCAGCCCACGTCCGTTGCCGGAC |
| **chrBC123Af** | TTGGCAACCCCTGCGGACGCACCGCCGGATGCGTTGGGC |
| **chrBC123Ar** | GCCCAACGCATCCGGCGGTGCGTCCGCAGGGGTTGCCAA |
| **chrBR175Af** | TTGGATTACCAGGCCGCTACCTGGGCCACTCGC |
| **chrBR175Ar** | GCGAGTGGCCCAGGTAGCGGCCTGGTAATCCAA |
| **chrBR180Af** | CGTACCTGGGCCACTGCCGCACGTCCTTGGGTT |
| **chrBR180Ar** | AACCCAAGGACGTGCGGCAGTGGCCCAGGTACG |
| **chrBR182Af** | TGGGCCACTCGCGCAGCTCCTTGGGTTGATCGG |
| **chrBR182Ar** | CCGATCAACCCAAGGAGCTGCGCGAGTGGCCCA |
| **chrBR187Af** | CGTCCTTGGGTTGATGCGCTCGCCAGCGCATGG |
| **chrBR187Ar** | CCATGCGCTGGCGAGCGCATCAACCCAAGGACG |
| **chrBR195Af** | AGCGCATGGCTGATCGCGCGCTTCATCGACCCG |
| **chrBR195Ar** | CGGGTCGATGAAGCGCGCGATCAGCCATGCGCT |
| **chrBR196Af** | GCCTGCGGGTCGATGAAGGCCCGGATCAGCCATGCGC |
| **chrBR196Ar** | GCGCATGGCTGATCCGGGCCTTCATCGACCCGCAGGC |
| **chrBH229Af** | GGCGCGACGTTCAGCGCTGTCGGCAGCCGTGTC |
| **chrBH229Ar** | GACACGGCTGCCGACAGCGCTGAACGTCGCGCC |
| **ChrBA241Rf** | CGTTCGAGGTCCTGGCGCGCAGCTTTGGGCTGGAACA |
| **ChrBA241Rr** | TGTTCCAGCCCAAAGCTGCGCGCCAGGACCTCGAACG |
| **ChrBG244Rf** | GTCCTGGCGGCGAGCTTTCGTCTGGAACAGCCCGCCATC |
| **ChrBG244Rr** | GATGGCGGGCTGTTCCAGACGAAAGCTCGCCGCCAGGAC |
| **chrBR258Af** | ATTGGCCTTGTGGTGGCTTACCTCGACGTGGGC |
| **chrBR258Ar** | GCCCACGTCGAGGTAAGCCACCACAAGGCCAAT |
